# Differential Responses of Stomata and Photosynthesis to Elevated Temperature in Two Co-occurring Subtropical Forest Tree Species

**DOI:** 10.3389/fpls.2018.00467

**Published:** 2018-04-10

**Authors:** Guilin Wu, Hui Liu, Lei Hua, Qi Luo, Yixue Lin, Pengcheng He, Shiwei Feng, Juxiu Liu, Qing Ye

**Affiliations:** ^1^Key Laboratory of Vegetation Restoration and Management of Degraded Ecosystems, South China Botanical Garden, Chinese Academy of Sciences, Guangzhou, China; ^2^College of Life Science, University of Chinese Academy of Sciences, Beijing, China; ^3^Guangdong Provincial Key Laboratory of Applied Botany, South China Botanical Garden, Chinese Academy of Sciences, Guangzhou, China; ^4^College of Natural Resources and Environment, South China Agricultural University, Guangzhou, China

**Keywords:** functional traits, gas exchange, subtropical tree species, warming, water deficit

## Abstract

Global warming could increase leaf transpiration and soil evaporation, which can potentially cause water deficit to plants. As valves, leaf stomata can control plant water loss and carbon gain, particularly under water stress conditions. To investigate the responses of stomata to elevated temperature in *Schima superba* and *Syzygium rehderianum*, two co-occurring subtropical forest dominant tree species, functional traits related to gas exchange, stomatal anatomy, and drought resistance were measured under control and warming environment (ca. 2°C higher). We found that leaf water potential at both predawn and midday significantly decreased for the two species grown under warming conditions compared with those grown in the control environment. Warming resulted in significant decrease of stomatal size in *S. rehderianum*, but had no obvious effect on that of *S. superba*. By contrast, stomatal density of *S. superba* significantly decreased under warming conditions, while non-significant change was observed for *S. rehderianum*. In addition, warming significantly reduced photosynthetic rate, stomatal conductance, and stomatal sensitivity to leaf water potential of *S. superba*, but had non-significant effects on those of *S. rehderianum*. Overall, our results demonstrated that, confronting water deficit caused by elevated temperature, the two co-occurring subtropical tree species responded differently through the adjustment of stomatal morphology and photosynthetic function. Consequently, *S. rehderianum* was able to maintain similar carbon assimilation as under control environment, while *S. superba* showed a decrease in carbon gain that might bring adverse effect on its dominancy in subtropical forest community under future climate change.

## Introduction

Recent predictions of global climate change suggest that temperature would continue to increase in the future 100 years (Christensen et al., 2007; Intergovernmental Panel on Climate Change [IPCC], 2014). Regardless of future changes in precipitation, increased temperature would increase soil evaporation and leaf transpiration, thus resulting in potential water stress on plants. Over the last decade, field observational studies across forests worldwide have documented changes in forest structure and tree mortality, which have been attributed to climate warming and severe droughts (Adams et al., 2009; Williams et al., 2012; Anderegg et al., 2013; Zhou et al., 2013; McIntyre et al., 2015). However, the physiological mechanisms that govern the growth and survival of plants by climate warming are not well understood. It is known that stomata can function as valves to control the balance of water loss and carbon gain in plants, which primary determine key physiological processes of plants (Farquhar et al., 1980; Luomala et al., 2005). Thus, a better understanding of stomatal response to climate warming may provide deeper insights into the acclimation and adaptation of plants under global climate change scenarios.

It has been shown that stomatal traits are strongly affected by elevated temperature. Recently studies have indicated that plants acclimate to a warmer environment by adjusting their optimum for growth and photosynthesis (Way and Yamori, 2014; Scafaro et al., 2017; Drake et al., 2018), and the relationship between stomatal conductance and water potential and vapor pressure deficit (VPD; Mott and Peak, 2010; Marchin et al., 2016; Urban et al., 2017). But understanding of the effects of elevated temperature on anatomical traits of stomata, which are linked to gas exchange traits, is limited for tropical and subtropical trees. In general, plants may respond quickly to short-term temperature changes by adjusting the openness/closure of the stomatal pores (Zhou et al., 2010). By contrast, long-term (annual to decadal) environmental changes such as climate warming may affect stomatal aperture size, stomatal density, and the pattern of stomatal distribution in leaves (McElwain et al., 1999; Yan et al., 2017). As a result, the change in stomatal density may modify the number of sites available for gas exchange per unit leaf area, while change in stomatal size may modify stomatal conductance by changing stomatal pore area through which CO_2_ can enter the leaf (Maherali et al., 2002; Franks and Farquhar, 2007). Although it has been shown that elevated temperature may have effects on both stomatal size and density (Beerling and Chaloner, 1993; McElwain et al., 1999; Yan et al., 2017), the results have been quite variable. For instance, in contrast to increases of leaf stomatal density in response to warming (Fraser et al., 2009; Hill et al., 2014), decreases in stomatal density were also observed (Luomala et al., 2005). Besides, some studies showed no change in stomatal density upon warming treatment (Zheng et al., 2013; Kivimäenpää et al., 2017). Similarly, warming had diverse effects on stomatal size across species, such that either increases (Ferris et al., 1996; Zheng et al., 2013), decreases (Zhang et al., 2010), or no change (Apple et al., 2000; Hill et al., 2014) in stomatal size were reported. Therefore, in parallel to investigating the change in stomatal size and density under elevated temperature condition, monitoring the change in key physiological processes such as gas exchange and drought resistance may allow for a more comprehensive understanding of plant response and adaptation to warming.

In this study, we selected two co-occurring dominant species (*Schima superba* and *Syzygium rehderianum*) from a subtropical forest in southern China. As found in our previous studies at the same site (e.g., Zhu et al., 2013; Li et al., 2015), the canopy of *S. superba* can reach 8–10 m, while *S. rehderianum* is a shrub-like species that usually grows 3–5 m high in this forest. Open top chamber (OTC) facilities were employed such that treated plants experienced ca. 2°C higher atmosphere temperature compared with the control plants. Stomatal anatomical traits (stomatal size and density) were determined, in parallel to measurements of physiological traits that are related to gas exchange [photosynthetic rate, stomatal conductance, and water use efficiency (WUE)] and plant water status (leaf water potential at predawn and midday). Specifically, we asked: (i) how would warming affect stomatal anatomy of these two co-occurring dominant tree species in subtropical forests and (ii) would these two species respond differently to warming in terms of controlling water balance and carbon assimilation through stomatal regulation?

## Materials and Methods

### Study Site

This study was carried out at the Dinghushan Biosphere Reserve (23° 09′N–23° 11′N, 112° 30′E–112° 33′E; DBR), with an area of 1155 ha. DBR is located in the middle of Guangdong Province in southern China. It is characterized by a typical subtropical monsoon climate, with mean annual temperature ca. 21°C, ranging from 12.6°C in the coldest month (January) to 28.0°C in the warmest month (July). The mean annual precipitation is ca. 1900 mm, of which about 80% occurs in the wet season (through April to September).

### Experimental Design and Treatments

Six pools were constructed using bricks and concrete. The pools were 3 m × 3 m in area, 0.8 m in depth, and 0.1 m high above the ground. Three of the six pools were sealed by transparent glass to form OTCs (3 m in height), and the rest were used as controls. Both control pools and OTCs receive only ambient precipitation. The chambers were heated by forced air blown over closed-loop resistance wires surrounding the OTCs at 1.5 m in height to simulate warming environment. Sensors were installed inside and outside the OTCs to monitor air temperature (HMP 155A, Vaisala, Finland), soil profile temperatures (CT 109, Campbell, United States), and volumetric soil water content (CS616, Campbell, United States) at 5 cm depth. Each meteorological observation was connected with a data logger (CR1000, Campbell, United States) to record data every 1 h. The chambers were heated year-round, both day and night, resulting in ca. 2°C higher of air temperature in the OTCs compared with the non-heated control pools.

Seedlings (1-year-old) of *S. superba* and *S. rehderianum* were collected, with six individuals of each study species transplanted into each of the OTCs and the control pools. Functional traits were measured after 1 year warming treatment.

### Predawn and Midday Leaf Water Measurements

We determined plant water status by measuring leaf water potential at predawn (Ψ_pd_) and midday (Ψ_md_) using a pressure chamber (PMS, Corvallis, OR, United States). Measurements were carried out between 06:30 and 07:30 for Ψ_pd_ and between 13:00 and 14:00 for Ψ_md_. A minimum of nine fully expanded mature leaves from three individuals per species in each OTC or the control pool were sampled for leaf water potential measurements.

### Gas Exchange Measurement

Leaf gas exchange was measured between 9:00 and 11:00 using a portable photosynthesis system equipped with a CO_2_ injector (Li6400, Li-Cor, Lincoln, NE, United States). Based on preliminary trials, the photosynthetic photon flux density was set at 1500 μmol m^-2^s^-1^ to ensure that light-saturated photosynthesis rates were reached for the two study species. Ambient CO_2_ was maintained at 400 μmol mol^-1^ and leaf temperature at 25°C for all measurements. Before data were recorded, leaves were exposed to the above conditions for about 15 min to allow photosynthetic parameters to stabilize (Zotz and Mikona, 2003). Similar to the sampling way for leaf water potential measurements, nine fully expanded mature leaves from three individuals per species in each OTC or the control pool were selected, for the quantification of CO_2_ assimilation rate (*A*_n_) and stomatal conductance (*g_s_*). Leaf-level WUE was calculated as WUE = *A*_n_/*T*_r_, where *T*_r_ is the leaf transpiration rate.

To test the relationship between stomatal conductance and leaf water potential, stomatal conductance, and VPD, nine fully expanded mature leaves from three individuals per species in each OTC or the control pool were selected and marked. Photon flux density and ambient CO_2_ concentration were set at 1500 μmol m^-2^s^-1^ and 400 μmol mol^-1^, respectively, and the measurement was carried out at their respective ambient conditions (control plants at control temperature and warmed plants at elevated temperature). We periodically measured gas exchange of all the marked leaves from 9:00 to 14:00 at an interval of an hour, each rotation the same marked leaves were measured at three times. Simultaneously, we cut leaves near the marked leaves to measured leaf water potential of each individual at each rotation.

### Leaf Anatomic Traits’ Measurement

Stomatal anatomic traits were measured following the protocols described by Carins Murphy et al. (2012). In brief, cuticles were stained with diluted (c. 0.1%) aqueous toluidine blue, rinsed, mounted on microscope slides, covered with a cover slip, gently pressured with fine-point tweezers, and then immediately observed under a light microscope (YS100, Nikon, Tokyo, Japan). Stomatal density (*D*) and guard cell width (*W*) and length (*L*) were measured using an image analysis software (OPTPro 2012 4.0, Optec XTS20, Chongqing Optec Instrument, China). Stomatal size (*S*) was defined as guard length (*L*) multiplied by the total width (2*W*) of the guard cell pair (Franks et al., 2009).

### Statistical Analysis

Trait values of the control and warming-treated plants of the two study species were compared using nested ANOVA. The difference in stomatal conductance to leaf water potential sensitivity was tested using analysis of covariance (ANCOVA). All statistical analyses were performed using SPSS 13.0 (SPSS Inc., Chicago, IL, United States).

## Results

### Environmental Variables

**FIGURE 1:**
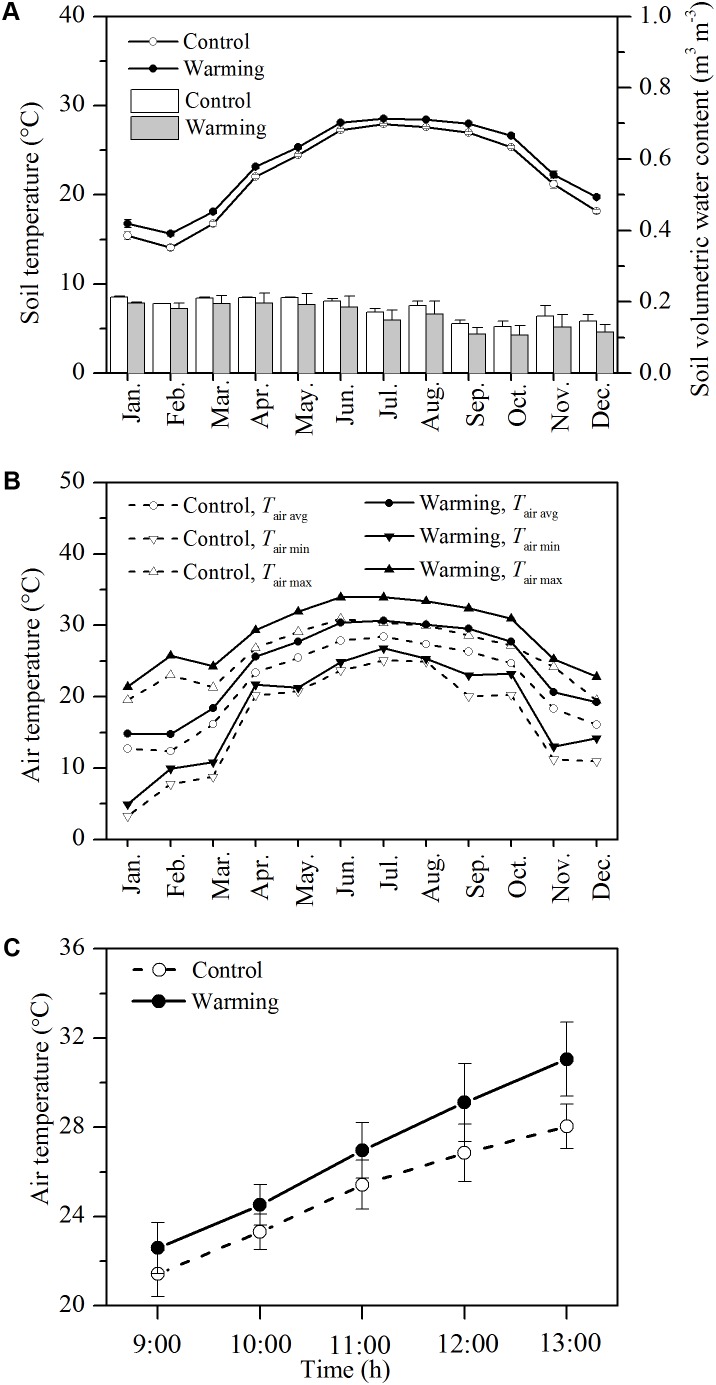
Monthly mean soil temperature (lines) and volumetric soil water content **(A)**, daily mean air temperature during gas exchange measurement **(B)**, monthly maximum, minimum, and mean air temperature **(C)** in the control and warming OTCs. Values are mean ± SD (*n* = 3 OTCs).

The monthly air and soil temperatures at the warming environment were distinctly higher than those at the control environment. The mean soil temperature at 5 cm in depth was 1.14°C higher in the warming environment than in the control environment (**Figure 1A**, *P* < 0.01). The mean soil moisture was not significantly lower in the warming environment than control environment (**Figure 1A**, *P* > 0.05). Upon the warming treatment, the mean, maximum, and minimum monthly air temperatures were 2.53, 2.90, and 1.83°C higher in the warming environment than those in the control environment, respectively (**Figure 1B**, *P* < 0.01). During gas exchange measurement, the mean, maximum, and minimum air temperatures were 2.35, 3.12, and 1.17°C higher in the warming environment than those in the control environment, respectively (**Figure 1C**, *P* < 0.05).

### Predawn and Midday Leaf Water Potential

**FIGURE 2:**
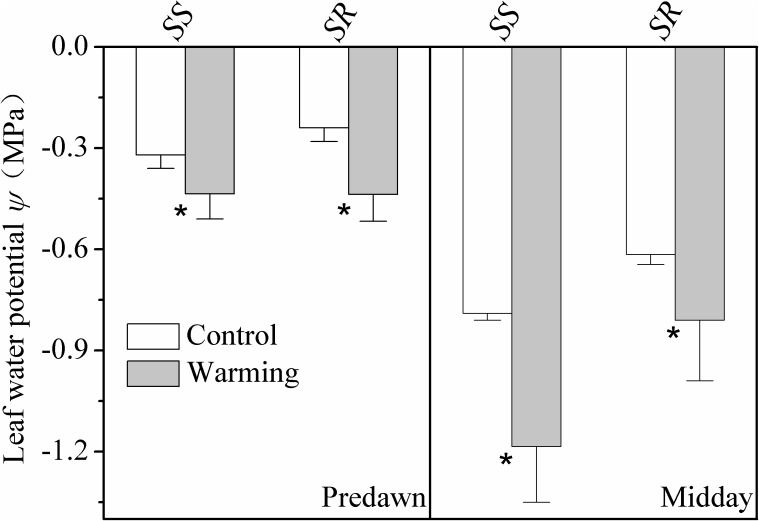
Leaf water potential at predawn (Ψ_pd_) and midday (Ψ_md_) for the two study species grown in both control and warming environments. Values are mean ± SD (*n* = 9 individual trees). ^∗^Indicates the significant difference at *P* < 0.05 (nested ANOVA). *SS* and *SR* represent *Schima superba* and *Syzygium rehderianum*, respectively.

Leaf water potential at predawn and midday of the warming plants was significantly lower (more negative) than plants grown in control condition for both *S. superba* and *S. rehderianum* (*P* < 0.05; **Figure 2**).

### Stomatal Size and Density

**FIGURE 3:**
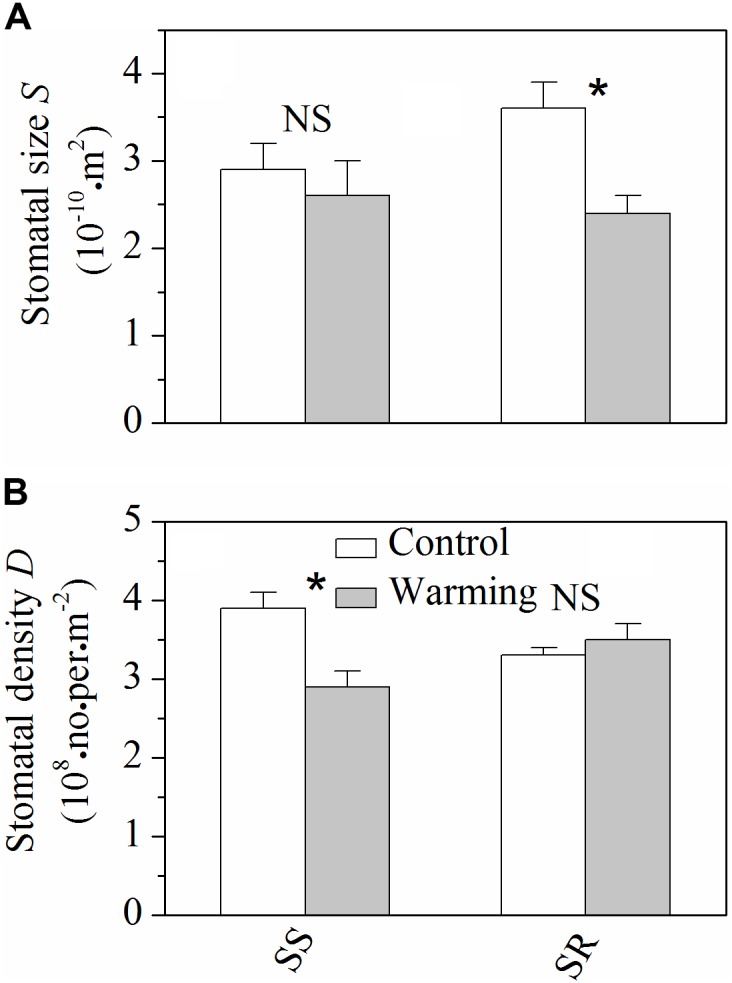
**(A)** Stomatal size (*S*) and **(B)** stomatal density (*D*) for the two study species grown in both control and warming environments. Values are mean ± SD (*n* = 9 trees). NS and ^∗^Indicate no significant difference and significant difference at *P* < 0.05 (nested ANOVA), respectively. *SS* and *SR* represent *Schima superba* and *Syzygium rehderianum*, respectively.

Warming resulted in significant decrease (*P* < 0.05) of stomatal size in *S. rehderianum*, but had no obvious effect (*P* > 0.05) on that of *S. superba* (**Figure 3A**). By contrast, stomatal density of *S. superba* significantly decreased (*P* < 0.05) under warming conditions, while non-significant change was observed for *S. rehderianum* (**Figure 3B**).

### Leaf Gas Exchange

**FIGURE 4:**
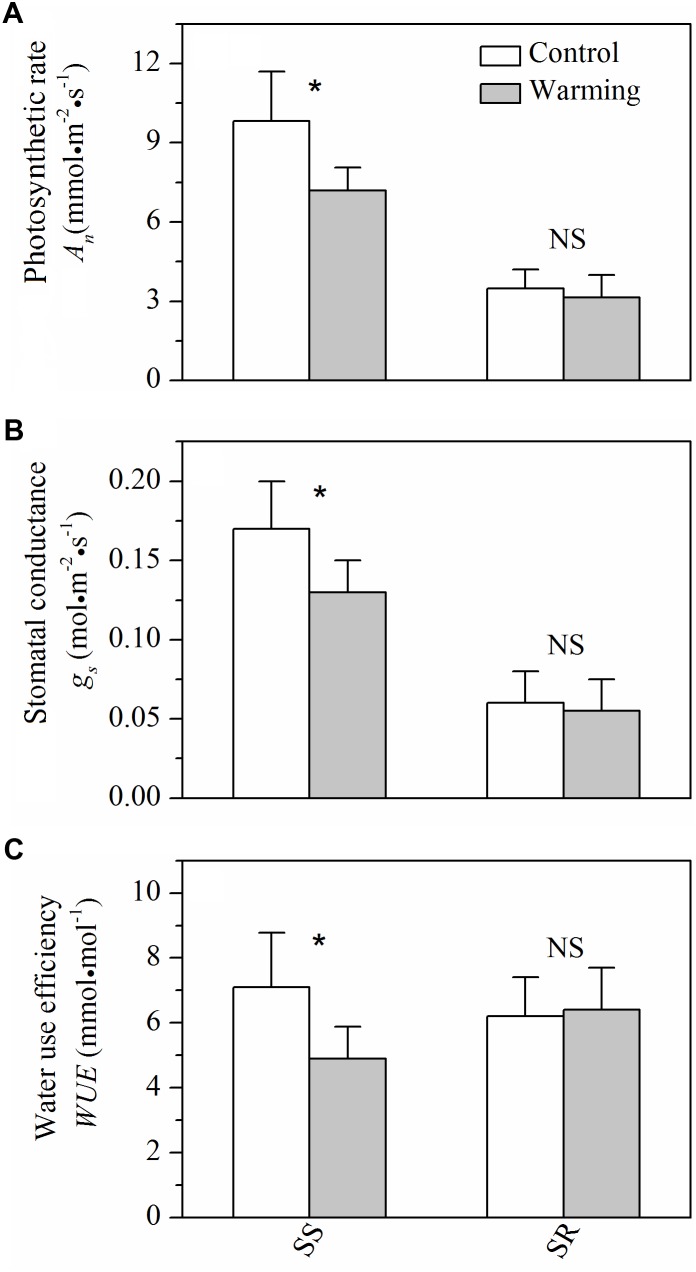
**(A)** Light-saturated photosynthetic rate (*A*_n_), **(B)** stomatal conductance (*g*_s_), and **(C)** water use efficiency (WUE) for the two study species grown in both control and warming environments. Values are mean ± SD (*n* = 9 trees). NS and ^∗^Indicate no significant difference and significant difference at *P* < 0.05 (nested ANOVA), respectively. *SS* and *SR* represent *Schima superba* and *Syzygium rehderianum*, respectively.

For gas exchange characteristics, warming caused significant decreases (*P* < 0.05) in photosynthetic rate, stomatal conductance, and WUE in *S. superba*, while no significant (*P* > 0.05) effects of warming on those of *S. rehderianum* were observed (**Figure 4**).

**FIGURE 5:**
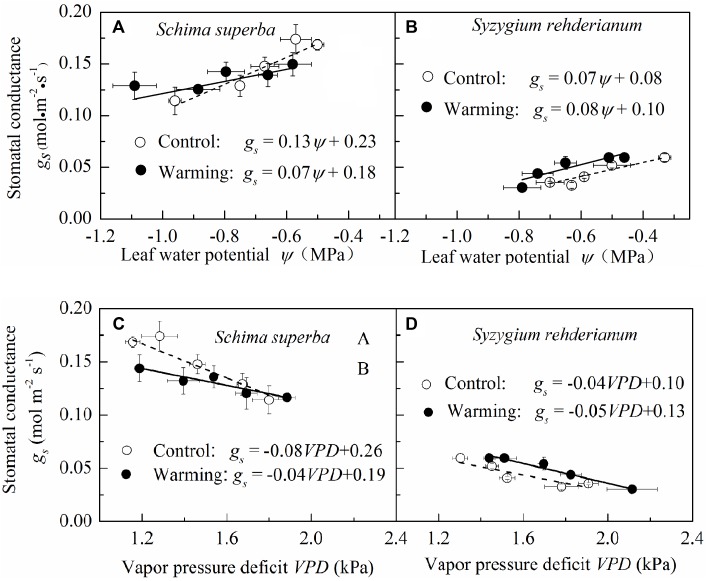
Relationship between stomatal conductance and leaf water potential **(A,B)** and vapor pressure deficit **(C,D)** for the two study species grown in both control and warming environments. Values are mean ± SE (*n* = 9 trees). Slope of stomatal conductance to leaf water potential represents stomatal sensitivity to leaf water potential. Open circle (○) and filled circle (•) represent control and warming environments, respectively.

To detect the sensitivity of stomatal conductance to leaf water potential and VPD, we fitted the relationship between stomatal conductance, leaf water potential, and VPD with linear models. We found that, for the two species, stomatal conductance was positively correlated with leaf water potential and negatively correlated with VPD both in warming and control conditions. However, both stomatal sensitivities to leaf water potential and to VPD were significantly higher (*P* < 0.05) in control than in warming environment for *S. superba*, as indicated by a steeper slope of the regression line, while the difference of stomatal sensitivity to leaf water potential and VPD was not significant (*P* > 0.05) for *S. rehderianum* (**Figure 5**).

## Discussion

We found in this study that the two species showed contrasting stomatal responses to elevated temperature. Warming significantly affected stomatal morphology (stomatal size or density) and its sensitivity to water deficit in both study species. *S. superba* employed a conservative strategy by reducing stomatal density but keeping a constant stomatal size, which can potentially prevent excessive water loss, but at the cost of reducing carbon gain (i.e., decrease in photosynthetic rate). By contrast, *S. rehderianum* was able to balance water loss and carbon gain through reducing stomatal size without affecting stomatal density, thus allowing the maintenance of photosynthetic rate under warming conditions.

### Warming Induced Changes in Stomatal Anatomic and Gas Exchange Traits

In general, plants with lower stomatal density would prevent water from excessive loss (Hepworth et al., 2015), but leaf stomatal conductance and photosynthetic rate might be reduced correspondingly (Xu and Zhou, 2008; Yoo et al., 2010; Upadhyay et al., 2013). For example, leaves with lower stomatal density have fewer pores through which CO_2_ can enter, resulting in potential lower stomatal conductance and photosynthesis rates (Tanaka et al., 2013). Furthermore, smaller size of stomata would decrease CO_2_ diffusion into the leaf (Parkhurst, 1994), because its conductance is proportional to the square of the effective radius of the pore (Maherali et al., 2002). However, smaller size of stomatal would allow plants to respond to environment change more promptly, thus potentially enhancing CO_2_ assimilation (Hetherington and Woodward, 2003; Franks et al., 2009). Such an adjustment is advantageous especially under water deficit conditions. For instance, Spence et al. (1986) reported that smaller size of stomata may allow the pores remaining open under drought stress, leading to the maintenance of photosynthesis. Interestingly, Hughes et al. (2017) showed that the decrease of both stomatal density and size in *Hordeum vulgare* reduced stomatal conductance and photosynthetic rate under well-watered conditions, but displayed non-significant effect under drought stress. Therefore, it is likely that the advantage of smaller size of stomata counteracted the negative effect on plant photosynthesis caused by lower stomatal density. In the present study, warming caused water deficit in both species, and we observed decreases of stomatal conductance and photosynthetic rate in *S. superba*, which might be due to the decrease of stomatal density under warming conditions; whereas *S. rehderianum* was able to maintain stomatal conductance and photosynthetic rate, probably owing to its reduced stomatal size in response to warming.

Apart from the adjustment of stomatal structure to warming, the two species may have different optimum growth temperatures, because *S. superba* is a canopy tree, while *S. rehderianum* is a shrub-like species growing in the understory. Therefore, the different ecological niche (e.g., canopy height) might result in different light sensitivity of the two species during the ontogeny, which might be one of the reasons for the observed contrasting responses in photosynthetic rate of the two species to warming (Way and Yamori, 2014; Slot and Winter, 2017a,b, 2018). In addition, warming might have different effects on Rubisco content (Scafaro et al., 2017) and mesophyll conductance (Qiu et al., 2017), leading to different changes in photosynthetic rate for different plants, which deserves further investigation for the two study species.

We noted that although the two species responded differently to warming through the adjustment of stomatal morphology and photosynthetic rates, non-significant differences in plant height and base diameter for the two species were observed after 1 year warming treatment (Supplementary Figure S1). On the one hand, both *S. superba* and *S. rehderianum* are slow-growing species with a slow “return on investment” (Zhu et al., 2013), and hence, a longer term warming treatment might be needed to investigate the effects on growth for the two species. On the other hand, as shown in a number of studies, warming might cause a decrease of dark respiration rate (Yamori et al., 2014; Slot and Winter, 2017a), which in turn results in less carbon loss in maintenance processes and more carbon can be retained for growth. This also needs to be investigated in future studies.

### Warming Induced Changes in Stomatal Sensitivity to Leaf Water Potential and Vapor Pressure Deficit

Stomatal sensitivity to leaf water potential and VPD reflects the balance of carbon gain and water use in plants (Fredeen and Sage, 1999; Buckley, 2005; Martínez-Vilalta and Garcia-Forner, 2017). High stomatal sensitivity to leaf water potential and VPD indicates that plants can effectively regulate their stomata aperture according to leaf water status. Such a strategy represents an advantage of plants in adaptation to water deficit, in which plants can fully open stomata when water status is well and close them when water status is unfavorable (Williams and Ehleringer, 2000; Addington et al., 2004; McDowell et al., 2008). By contrast, plants with low stomatal sensitivity to leaf water potential and VPD may have advantage when water condition is favorable, but readily suffer from water stress because they lack the “smart” regulation manner in preventing excessive water loss (Dai et al., 1992; Domec and Johnson, 2012; Klein, 2014). We found, in the present study, a significant decrease of stomatal sensitivity to leaf water potential and VPD in *S. superba*, but non-significant change in that of *S. rehderianum*. Our results indicate that stomatal adjustment in *S. rehderianum* is more advantageous than that of *S. superba* under water deficit environment caused by elevated temperature.

## Conclusion

Our study clearly demonstrated that, *S. rehderianum* and *S. superba*, the two co-occurring subtropical forest dominant tree species showed deferential responses to elevated temperature in terms of stomatal structure and photosynthetic function. The decreased stomatal size and constant stomatal density of *S. rehderianum* allowed the maintenance of carbon assimilation rate, WUE, and high stomatal sensitivity to leaf water potential, while the converse was true for *S. superba*, which may, in a long term, imperil its dominancy in this subtropical forest community under future climate change.

## Author Contributions

GW and QY conceived the idea. GW and LH conducted the experiments. GW analyzed the data. QY, HL, and GW wrote the manuscript. All authors helped in drafting the manuscript and gave essential input to the work.

## Conflict of Interest Statement

The authors declare that the research was conducted in the absence of any commercial or financial relationships that could be construed as a potential conflict of interest.
