# Supplementary material for: Differential Responses of Stomata and Photosynthesis to Elevated Temperature in Two Co-occurring Subtropical Forest Tree Species
Source: Front Plant Sci. 2018 Apr 10;9:467. doi: 10.3389/fpls.2018.00467 (PMC5928911; doi:10.3389/fpls.2018.00467)
Supplement: Supplementary file 1 [file Image_1.pdf]

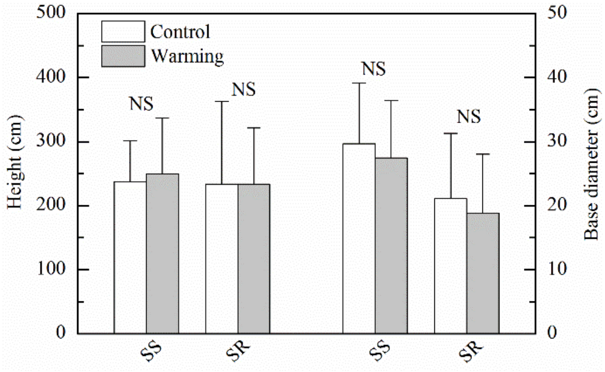

Fig. S1 Height and Base diameter for the two study species grown in both control and warming environments. Values are mean ± SD. NS indicates non-significant difference at $P < 0.05$ (nested ANOVA). *SS* and *SR* represent *Schima superba* and *Syzygium rehderianum*, respectively.
